# Prevalence and localization of nocturnal epileptiform discharges in mild cognitive impairment

**DOI:** 10.1093/braincomms/fcad302

**Published:** 2023-11-08

**Authors:** Rosario Ciliento, Klevest Gjini, Kevin Dabbs, Bruce Hermann, Brady Riedner, Stephanie Jones, Safoora Fatima, Sterling Johnson, Barbara Bendlin, Alice D Lam, Melanie Boly, Aaron F Struck

**Affiliations:** Department of Neurology, University of Wisconsin-Madison, Madison, WI 53726, USA; Department of Neurology, University of Wisconsin-Madison, Madison, WI 53726, USA; Department of Neurology, University of Wisconsin-Madison, Madison, WI 53726, USA; Department of Neurology, University of Wisconsin-Madison, Madison, WI 53726, USA; Department of Psychiatry, University of Wisconsin-Madison, Madison, WI 53719, USA; Department of Psychiatry, University of Wisconsin-Madison, Madison, WI 53719, USA; Department of Neurology, University of Wisconsin-Madison, Madison, WI 53726, USA; Department of Medicine, University of Wisconsin, Madison, WI 53705, USA; Department of Medicine, University of Wisconsin, Madison, WI 53705, USA; Department of Neurology, Harvard Medical School, Massachusetts General Hospital, Boston, MA 02115, USA; Department of Neurology, University of Wisconsin-Madison, Madison, WI 53726, USA; Department of Neurology, University of Wisconsin-Madison, Madison, WI 53726, USA; Department of Neurology, William S. Middleton Veterans Administration Hospital, Madison, WI 53705, USA

**Keywords:** Alzheimer’s disease, mild cognitive impairment, EEG spikes, source localization, neuropsychological assessment

## Abstract

Recent evidence shows that identifying and treating epileptiform abnormalities in patients with Alzheimer’s disease could represent a potential avenue to improve clinical outcome. Specifically, animal and human studies have revealed that in the early phase of Alzheimer’s disease, there is an increased risk of seizures. It has also been demonstrated that the administration of anti-seizure medications can slow the functional progression of the disease only in patients with EEG signs of cortical hyperexcitability. In addition, although it is not known at what disease stage hyperexcitability emerges, there remains no consensus regarding the imaging and diagnostic methods best able to detect interictal events to further distinguish different phenotypes of Alzheimer’s disease. In this exploratory work, we studied 13 subjects with amnestic mild cognitive impairment and 20 healthy controls using overnight high-density EEG with 256 channels. All participants also underwent MRI and neuropsychological assessment. Electronic source reconstruction was also used to better select and localize spikes. We found spikes in six of 13 (46%) amnestic mild cognitive impairment compared with two of 20 (10%) healthy control participants (*P* = 0.035), representing a spike prevalence similar to that detected in previous studies of patients with early-stage Alzheimer’s disease. The interictal events were low-amplitude temporal spikes more prevalent during non-rapid eye movement sleep. No statistically significant differences were found in cognitive performance between amnestic mild cognitive impairment patients with and without spikes, but a trend in immediate and delayed memory was observed. Moreover, no imaging findings of cortical and subcortical atrophy were found between amnestic mild cognitive impairment participants with and without epileptiform spikes. In summary, our exploratory study shows that patients with amnestic mild cognitive impairment reveal EEG signs of hyperexcitability early in the disease course, while no other significant differences in neuropsychological or imaging features were observed among the subgroups. If confirmed with longitudinal data, these exploratory findings could represent one of the first signatures of a preclinical epileptiform phenotype of amnestic mild cognitive impairment and its progression.

## Teaser

In this exploratory research, Ciliento *et al*., using high-density EEG overnight, detected EEG spikes in 46% of patients with amnestic mild cognitive impairment without any history of seizures versus 10% in controls. Complemented by MRI and neuropsychology, no significant morphological and cognitive differences emerged between patients with and without spikes.

## Graphical Abstract

**Graphical Abstract:**
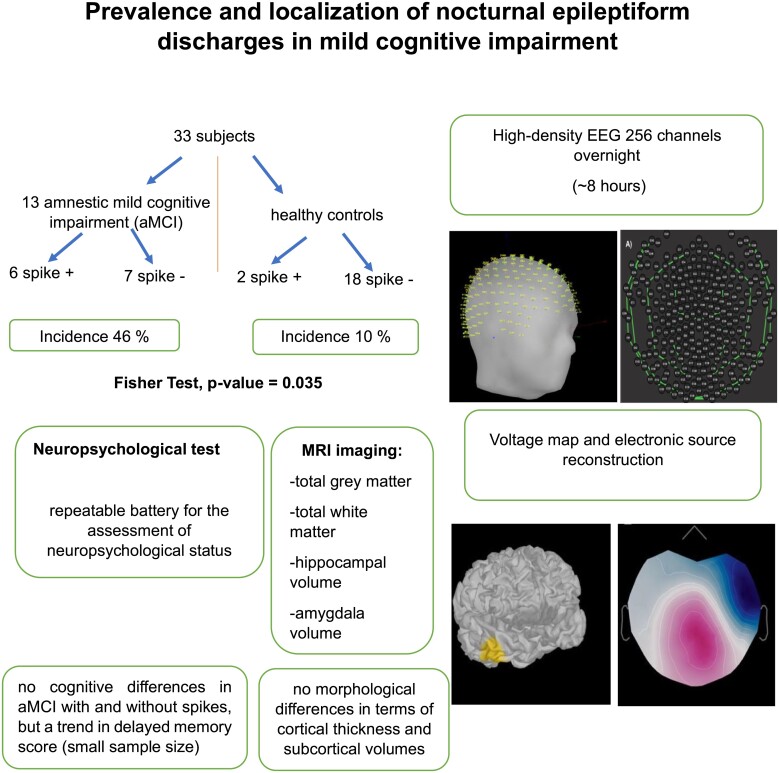

## Introduction

Alzheimer’s disease is the sixth leading cause of death among USA adults with 6.2 million Americans living with Alzheimer’s disease^1^; therefore, disease-modifying treatments are needed to ameliorate the cognitive decline in patients with Alzheimer’s disease. A potential point for intervention is identifying and treating patients with epileptiform abnormalities. From the earliest descriptions of Alzheimer’s disease, it was noted that there was an increased incidence of seizures.^2^ Furthermore, the incidence of seizures and development of epilepsy show a peak in the aging population (>65 years of age)^3^ that is only partially explained by increases in pertinent aetiologies such as cerebral vascular disease and brain tumours.^4^ It is unclear if the remaining increased risk is related to the direct result of the accumulation of pathological proteins as seen in neurodegenerative disorders or if normal brain aging with generalized atrophy and accompanying neuro(dys)plasticity is pro-epileptic by itself. Most data suggest that the Alzheimer’s disease-related pathogenic cascade is more relevant than brain aging as patients who carry the *APP*, *PSEN1* or *PSEN2 gene* mutations linked to autosomal dominant early onset Alzheimer’s disease have, if anything, a greater risk of seizures (∼28%) than those with later onset Alzheimer’s disease.^5^

In particular, the disruption of the balance between the pre- and post-synaptic activities related to Alzheimer’s disease pathology^6,7^ results in an increase in neuronal excitability.^7^ This, in turn, contributes to the accumulation of extracellular amyloid-beta(AB) oligomers, tau-related neurodegeneration, synaptic loss, gliosis and hastened cognitive decline through a positive feedback loop.^7^ Identifying early markers for this ‘epileptiform Alzheimer’s disease phenotype’ with increased neuronal hyperexcitability could provide targets for interventions to interrupt this pathological cascade.^6,7^

Epileptiform discharges or EEG spikes are interictal non-invasive markers of hyperexcitability in patients with epilepsy. The extent of epileptiform discharges within Alzheimer’s disease is not entirely clear. Studies using EEG and Magnetoencephalography (MEG) show that up to 40% of patients with Alzheimer’s disease exhibit epileptiform spikes,^8-11^ compared with only approximately 5–10% of cognitively normal age-matched subjects. Fewer patients with Alzheimer’s disease who have interictal spikes go on to exhibit overt clinical seizures (10–22%).^3,8^ However, Alzheimer’s disease patients with epileptiform spikes have a faster cognitive decline and reduced survival.^12,13^

Determining when epileptiform activity first develops would provide an early window for treatment and establish the relationship of epileptiform activity to the accumulation of amyloid, tau and neurodegeneration. Early intervention is key for anti-amyloid therapies, and the same may hold true for the epileptiform phenotype. Indeed, a recent clinical trial using levetiracetam in early Alzheimer’s disease showed benefit only in the subgroup of patients with EEG spikes.^14^

However, there is currently no gold-standard method to detect epileptiform spikes in patients on the Alzheimer’s disease spectrum. In this study, we propose the use of prolonged high-density EEG (HD-EEG): this technique has expanded temporal lobe coverage that could potentially help in detecting smaller amplitude nocturnal spikes, especially if they originate from deep structures like amygdala, hippocampus or entorhinal cortex, sites involved early in the disease course of Alzheimer’s disease. In HD-EEG, the ability to detect subtle temporal lobe spikes is similar to MEG, but unlike MEG, HD-EEG can be performed overnight to capture the highest yield time periods in slow-wave sleep. Thus, HD-EEG combines the improved sensitivity of MEG with the ability to have overnight recording of standard-density EEG.

Specifically, our goal is to determine the incidence of EEG spikes using overnight 256-channel HD-EEG on an earlier-stage disease cohort: patients with amnestic mild cognitive impairment (aMCI). Additionally, structural neuroimaging with MRI and neuropsychological assessment were collected. The primary outcome was the incidence of EEG spikes between patients with aMCI (*n* = 13) and age-matched controls [healthy control (HC)] (*n* = 20). Then, using electronic source imaging, we localized the cortical origin sites of electrical activity during nocturnal epileptiform discharges. In the end, we evaluated the differences in cognitive performance and cerebral atrophy between aMCI with EEG spikes (*n* = 6) and aMCI without EEG spikes (*n* = 7) and healthy age-matched controls (*n* = 18).

## Materials and methods

### Standard protocol approvals, registrations and patient consents

The Institutional Review Board of the University of Wisconsin and William S. Middleton Veterans Hospital approved all study procedures. All research was performed in accordance with relevant guidelines and regulations. Informed consent was obtained for all participants.

### Study population

Participants for this investigation included older veterans from the William S. Middleton VA and adults from the Wisconsin Alzheimer’s Disease Research Center with known or suspected aMCI (*n* = 13). A non-impaired control group (*n* = 20) was recruited and matched to the aMCI group for age, gender, race and educational status. *ApoE4* carrier status was determined for all participants (Table 1).

**Table 1:** Demographic features, spikes' distribution during sleep and sleep parameters

Characteristic	aMCI (*n* = 13)	Controls (*n* = 20)	*P*-value
Age, years	72 ± 7	72 ± 6	0.9508
Education, years	14	14	0.27
Male sex, *n* (%)	11 (84)	18 (90)	0.6433
Race White, *n* (%)	12 (92)	18 (90)	1
Race Indian native/Alaskan, *n* (%)	1 (8)	2 (10)	1
ApoE ɛ4 carrier, *n* (%)	9 (70)	4 (21)	0.0047
Spike prevalence (%)	46	10	0.035
Spike amplitude	43.7 ± 15.1	31.2 ± 12.3	0.1691
**EEG spikes during sleep stages**			
Awake, *n*	0	1	
N1, *n*	0	0	
N2, *n*	5	1	
N3, *n*	2	0	
REM, *n*	1	0	
**Sleep macrostructure**			
TST, m	341 ± 103	319 ± 70	0.678
SE (%)	72 ± 15	71 ± 14	0.8392
N1, m	13 ± 8	16 ± 11	0.386
N2, m	62 ± 14	63 ± 11	0.8682
N3, m	5 ± 5	4 ± 4	0.8798
REM, m	17 ± 7	16 ± 5	0.6177

SE, sleep efficiency; TST, total sleep time.

General inclusion criteria included age > 50, no depression [Center for Epidemiologic Studies Depression scale(CES-D) < 15] or anxiety (Spielberger State Anxiety Score < 55), English as the primary language and right handed. The aMCI group met formal aMCI criteria^15^ [no dementia, self-complaint or informant complaint of memory impairment, normal physical functioning, age and education-adjusted performance that was 1–1.5 SDs below normal defined objective impairment] based on formal neuropsychological and clinical evaluation. The control group had no known or suspected cognitive impairment.

Exclusion criteria included dementia (mini-mental status exam score < 25), lacked consent capacity, presence of brain tumour, subdural haematoma, vascular dementia, normal pressure hydrocephalus, major psychiatric or sleep disorder, substance abuse, MRI contraindications or current use of medications that affect cognition.

Supplementary material and Supplementary Fig. 1 provide further details about the experimental timeline.

### Neuropsychological testing measures

The cognitive battery included age and educated adjusted measures of immediate and delayed memory, language, executive function and visuospatial skills from the Repeatable Battery for Neuropsychological Status (RBANS) (Table 2). The RBANS is a standard and widely used battery evaluating a range of cognitive domains that help to differentiate amnestic from non-amnestic forms of MCI and single domain from multi-domain classes of the disorder.^16,17^ In addition to addressing diagnostic issues, the tests of memory and executive function are predictive of conversion from MCI to Alzheimer’s disease. Episodic memory testing is particularly sensitive to early Alzheimer’s disease,^18^ and memory impairment was defined as from >−1 to −1.5 SDs below average.^19^ In addition, the mini-mental status exam was administered.^17^

**Table 2:** Neuropsychological testing measures

Test	aMCI with spikes (*n* = 6)	aMCI without spikes (*n* = 7)	Controls (*n* = 20)	*P*-value (Kruskal––Wallis)	*P*-value (U-test)
RBANS immediate memory	72.6 ± 14.6	78.7 ± 19.3	96.6 ± 9.9	0.0023	0.46
RBANS visuospatial	94 ± 17.1	83.8 ± 10.5	92 ± 9.8	0.1214	0.19
RBANS language	95.8 ± 16.7	92.2 ± 8	95.8 ± 6.3	0.57	0.66
RBANS attention	105.1 ± 17.5	95.1 ± 14.1	97.3 ± 11.7	0.43	0.27
RBANS delayed memory	63.5 ± 10.3	73.8 ± 20.1	99.8 ± 8.8	<0.001	0.25
RBANS total	81.6 ± 7.9	80 ± 11	94.1 ± 6.5	0.0004	1
**RBANS subscales**					
WRAT	65.1 ± 26	70.2 ± 11.3	51 ± 26.8	0.13	0.75
Trial making test A	47.8 ± 18.5	42.2 ± 28.5	50.9 ± 29.6	0.76	0.65
Trial making test B	36.8 ± 22.2	36.6 ± 33.8	49 ± 26.6	0.5	0.94
IM list learning	18.3 ± 2.2	18.1 ± 5.8	24.65 ± 4.1	0.001	0.4
IM story memory	11.5 ± 4.1	14.1 ± 5.5	17.2 ± 2.9	0.029	0.31
VS figure copy	16.3 ± 2.1	13.4 ± 3.5	15.7 ± 2.2	0.1218	0.10
VS line orientation	16.8 ± 1.9	16.8 ± 1	17.5 ± 2	0.49	0.84
Language picture naming	9.5 ± 0.8	10 ± 0	9.9 ± 0.4	0.08	0.38
Language semantic fluency	18 ± 6.3	15 ± 3.5	17.9 ± 3.6	0.27	0.42
Attention digit span	11.1 ± 2	10.7 ± 2.8	9.6 ± 2.9	0.28	0.83
Attention coding	41 ± 9.8	34.7 ± 9	41.3 ± 6.9	0.35	0.63
DM list recall	1.5 ± 1.7	2 ± 1.7	5.7 ± 2.2	0.0001	0.63
DM list recognition	16 ± 1.4	15.7 ± 5.9	19.2 ± 0.9	0.013	0.45
DM story recall	5 ± 2.9	5.4 ± 4.1	8.7 ± 1.5	0.0191	0.907
DM figure recall	5.3 ± 4.9	5.4 ± 3.9	11.5 ± 3.4	0.0016	0.97

DM, delayed memory; IM, immediate memory; VS, visuo spatial; WRAT, wide range achievement test.

### HD-EEG

A 256-channel HD-EEG was applied to each participant for overnight recording. The HD-EEG recording was 8 h long. EEG sampling was 500 Hz, referenced to the vertex electrode, using a Net Amps 300 amplifier and Net Station software (Electrical Geodesics Inc., Eugene, OR, USA). In Net Station, a first-order high-pass filter (0.1 Hz) was applied followed by a band-pass filter (Kaiser type, 1–50 Hz) applied in MATLAB (The MathWorks Inc., Natick, MA, USA).

### EEG review

EEG was downsampled to 200 Hz, and 1 Hz high pass filtering and 40 Hz low pass filtering were applied. Then, using MATLAB (The MathWorks, Inc., Natick, MA, USA), channels containing artefact associated with excessive noise or inconsistent contact with the scalp were visually identified and replaced with data interpolated from nearby channels using spherical splines in Net Station (Electrical Geodesic Inc.) and then average referenced to the mean voltage across all good channels.

**Figure 1:**
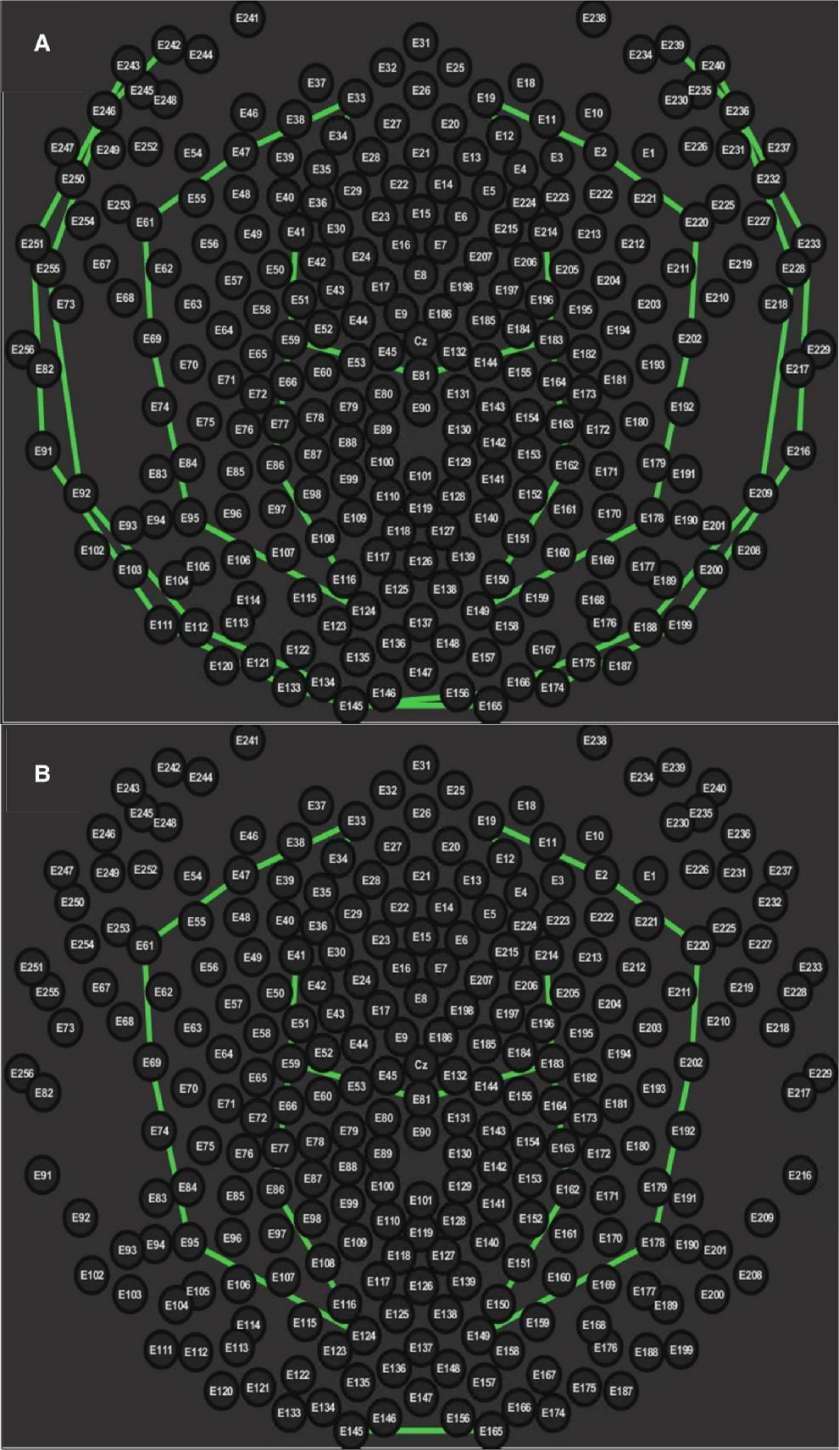
**Comparison of spatial coverage between 10- to 20- and bi-temporal extended HD-EEG montages.** (**A**) Clinical 10- to 20-bipolar EEG montage, overlaid on 256-electrode location. (**B**) Bi-temporal extended HD-EEG montages. Numbers correspond to electrode labels on EGI HD-EEG nets.

An extended, 40-bipolar channel montage was designed, to better visualize EEG activity from the anterior basal and inferior temporal and posterior brain regions. Figure 1 displays a comparison between clinical 10- to 20- and 41-channel HD-EEG montages.

All the data were first visually analysed by a board-certified neurologist (R.C.), who meticulously reviewed and manually marked any suspicious events for EEG spikes, without knowing the group allocation of the subjects. This analysis was conducted according to 2017 neurophysiology criteria.^20^ Subsequently, a blinded board-certified epileptologist (A.S.) conducted a thorough review of the initial marking. The second reviewer did not know the diagnosis of the subjects as well. During this step, additional events were marked, and others that did not meet the criteria for EDs were discarded. Additionally, electronic source imaging was performed, and non-physiological signals were discarded.

### MRI

MRI scans were acquired in the axial plane on a GE ×750 3.0-T scanner with an 8-channel phased array head coil (General Electric, Waukesha, WI, USA). 3D T_1_-weighted inversion recovery-prepared spoiled gradient echo scans were collected using the following parameters: inversion time/echo time/repetition time = 450 ms/3.2 ms/8.2 ms, flip angle = 12°, slice thickness = 1 mm no gap, field of view = 256 and matrix size = 256 × 256. MRI scans were processed using FreeSurfer version 6.0.0,^21^ and the operating system was CENTOS 6.

### Source space analysis

Two-second epochs were used for EEG spike electronic source imaging using BrainStorm (https://www.nitrc.org/projects/bst/)^22^ with a realistic boundary element method head model and a weighted minimum norm method as inverse solution. Individual MRI brain templates were used for all patients. The forward head model was created by applying a 256-lead placement pre-defined in BrainStorm software through realistic boundary element method option. The source space was constrained to the cortex, which was downsampled to 15 000 vertices. Source estimation was first achieved through minimum norm imaging, more specifically with Standardized low resolution brain electromagnetic tomography (sLORETA) method.^23^ Noise covariance was obtained using no NoiseModelling option (i.e. use of an identity matrix as noise covariance).

### Statistical analysis

Due to the small sample size, we decided to use non-parametric tests. We firstly identified two groups, patients with aMCI and HC. Fisher test was used to assess the distribution of sex, race, ApoE ɛ4 carrier status and spike incidence between aMCI (*n* = 13) and HC groups (*n* = 20). The Mann–Whitney U-test was used to assess group differences in age, years of education and spike amplitude and to investigate differences in neuropsychological test score between subjects with aMCI and HC.

Once we marked the EEG spikes, we identified three subcohorts: aMCI with spikes (*n* = 6), aMCI without spikes (*n* = 7) and controls (*n* = 20). Thus, Kruskal–Wallis test was applied to assess difference in neuroimaging values and neuropsychological scores.

We repeated the same analysis identifying the groups as follows: aMCI with spikes (*n* = 6), aMCI without spikes (*n* = 7), controls with spikes (*n* = 2) and controls without spikes (*n* = 18).

In addition, in order to investigate the potential effects of age, sex and education on cortical thickness and subcortical volumes across different groups, we applied a generalized linear model, assuming a Poisson distribution for the outcome variable, and the Kruskal–Wallis test to account for the non-normal distribution of the brain volume data.

For the neuropsychological data, after conducting the Kruskal–Wallis test, it became apparent that one of the variables significantly influenced the statistical differences. Subsequently, we performed a *post hoc* analysis using the Mann–Whitney U-test on the two subgroups, aMCI with spikes and aMCI without spikes, in order to eliminate the impact caused by the HCs who had higher scores by definition. Statistical analysis was performed with MATLAB (2020b version, Natick, MA, USA).

## Results

### Demographics

There were no statistically significant differences between the HCs (*n* = 20) and aMCI group (*n* = 13) in sex distribution, age, race and years of education. Higher prevalence of *ApoE4* allele carrier status was present in subjects with aMCI compared with HC (Table 1).

### Sleep parameters

Individual sleep parameters were examined in patients and controls (Table 1). At the group level, total sleep time (mean 341 ± 103 in aMCI versus 319 ± 70 in HC, *P* = 0.67) and sleep efficiency (72 ± 15% in aMCI versus 71 ± 14 in HC *P* = 0.83), the percentage of time spent in Stage N1 (13 ± 8% in aMCI versus 16 ± 11% in HC *P* = 0.386), N2 (62 ± 14% in aMCI versus 68 ± 11% in HC *P* = 0.8682) or N3 NREM sleep (5 ± 5% in aMCI versus 4 ± 4% in HC *P* = 0.8798) and REM sleep (17 ± 7% in aMCI versus 16 ± 5% in HC *P* = 0.6177) did not significantly differ between patients and controls. Table 1 shows additional information.

### EEG spikes

The primary outcome measure was the incidence of EEG spikes between the aMCI and control group.

We detected EEG spikes in 46% of subjects with aMCI (six of 13) and 10% of HC (two of 20). The difference is statistically significant, assessed with Fisher test (*P* = 0.035).

Table 1 specifies the prevalence, amplitude and sleep stage of EEG spikes for each participant.

The number of events was one to four per night in the aMCI group. Supplementary Table 1 exhibits the specific number of spikes per each subject. Four subjects showed spikes over the left hemisphere, two over the right and one bilaterally. In the HC group, the number of events was one to three per night and both subjects exhibited left hemisphere spikes.

According to 2017 neurophysiology criteria,^20^ the events were bi-phasic or tri-phasic events, with spiky morphology, clearly standing out of the background and associated with post-spike slow wave. Supplementary Figs 3 and 6 show examples of EEG spikes in a patient with aMCI and HC, respectively.

Using source reconstruction and voltage map, we were able to discard all the events that resembled an epileptiform discharge on the scalp EEG but lacked the appropriate distribution of negative and positive potentials on the scalp corresponding to a radial, oblique or tangential orientation of the source localized in the brain. Supplementary Figs 4 and 7 show an example of 2D topography of the voltage map at the time of the peak of the spikes in one patient with aMCI and one HC, respectively.

No significant differences in morphology were found between the EEG spikes in the two groups.

The mean amplitude of the events is 43.785 ± 15.15 µV in the MCI group and 31.285 ± 12.36 in the HC group.

Ultimately, the interictal events detected in our cohort were low-amplitude temporal spikes; most of them were detected during slow-wave sleep (NREM II and III). More specifically, one subject with aMCI had spikes in N2, N3 and REM, four subjects had spikes only during N2, and one subject had spikes only during N3. HCs showed spikes in wake (1) and N2 sleep (1).

### EEG spike electronic source imaging

A custom script was used to identify the negative amplitude peak of the spikes, which in turn was used for source reconstruction.

**Figure 2:**
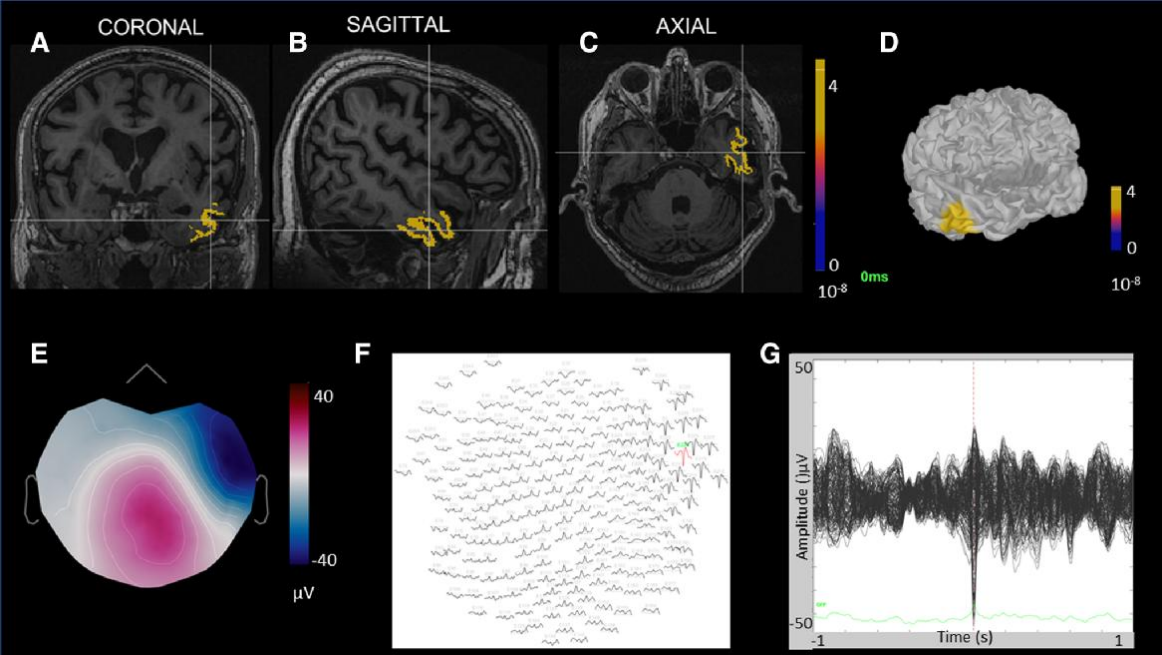
**Electronic source imaging in one patient with aMCI.** The enclosed area marked with the perpendicular lines identifies the sources at the time peak of one epileptiform discharge, shown on individual MRI viewer on (**A**) coronal, (**B**) sagittal and (**C**) axial view. (**D**) Cortical sources’ activation at spike’s time peak on 3D model, Standardized low resolution brain electromagnetic tomography (sLORETA) method. (**E**) Voltage topography map that shows the dipole at the spike’s peak. (**F**) Monopolar HD-EEG layout, in red the channel with the max amplitude at the spike’s peak. (**G**) Butterfly plot of the spike, −1000 to +1000 ms.

**Figure 3:**
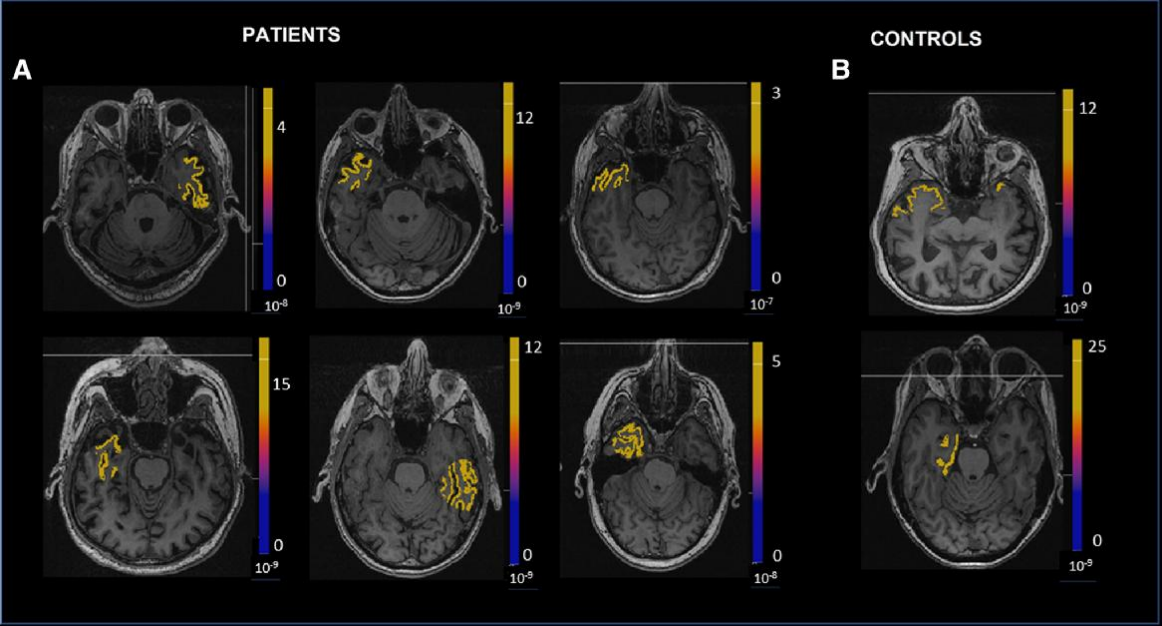
**Electronic source imaging in patients and controls.** Overview of sources at the time peak of one epileptiform discharge, shown on individual MRI viewer on axial view in (**A**) patients with aMCI and (**B**) HC.

The totality of the spikes showed the sources to be in the temporal lobes. Particularly, four localized to mTL, one to lateral neocortical temporal cortex, and two to lateral and mesial temporal cortex. HCs showed spikes in the left medial (1) and lateral regions (1). Figure 2 shows Electronic Source Imaging in one patient with aMCI. Figure 3 shows an overview of sources at the time peak of one epileptiform discharge, shown on individual MRI viewer on axial view in (**A**) patients with aMCI and (**B**) HC.

### ApoE ɛ4 carrier status

We conducted an analysis specifically examining differences in spikes among ApoE4 subjects in our sample. Our findings did not reveal any significant differences in terms of spikes between the *ApoE4* and non-*ApoE4* groups: aMCI with spikes = 4/6 (67%) and aMCI without spikes = 5/7 (71%), *P* = 1 (Supplementary Table 2 and Supplementary material).

### Neuropsychological assessment

There was a statistically significant difference between the aMCI and HC groups on the immediate (*P* < 0.001) and delayed memory (*P* < 0.001) tasks and total index score (*P* < 0.001).

According to inclusion criteria, a statistically significant difference was present between the aMCI and HC groups on the immediate (*P* < 0.001) and delayed memory (*P* < 0.001) RBAN subscores. No statistically significant differences were present between the aMCI and HC groups on the visuospatial skills, language and attention RBAN subscores.

**Figure 4:**
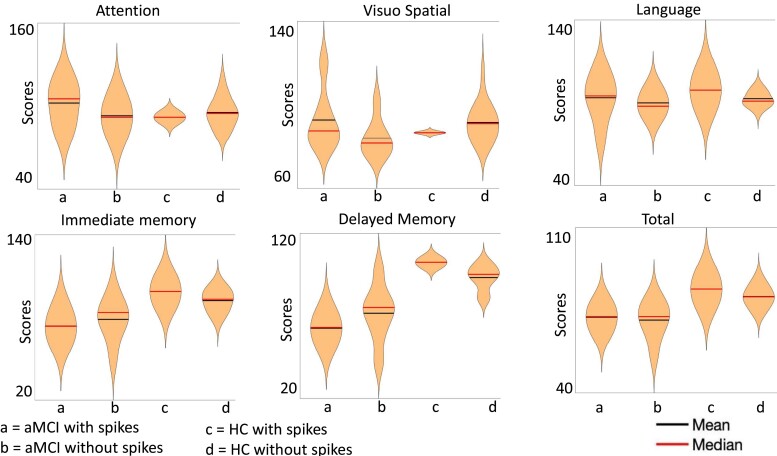
**Neuropsychological assessment.** Violin plots with scores comparison between patients with aMCI with spikes (violin plot 1), patients with aMCI without spikes (violin plot 2), HCs with spikes (violin plot 3) and HCs without spikes (violin plot 4). On the *Y*-axis, the scores. From left to right, top to bottom Attention, Visuo-Spatial skills, Language, Immediate Memory, Delayed Memory, Total RBANS score. No statistically significant differences were found between the aMCI groups with and without spikes and between the HC groups with and without spikes. Kruskal–Wallis test was used for analysis with all groups, U-test for the analysis with only two groups (aMCI with spikes versus aMCI without spikes). Attention Kruskal–Wallis *P*-value = 0.60; U-test *P*-value = 0.27. Visuospatial skills. Kruskal–Wallis *P*-value = 0.20; U-test *P*-value = 0.19. Language Kruskal–Wallis *P*-value = 0.65; U-test *P*-value = 0.27. Immediate memory. Kruskal–Wallis *P*-value = 0.005; U-test *P*-value = 0.46. Delayed memory. Kruskal–Wallis *P*-value < 0.001; U-test *P*-value = 0.25. Total RBANS score. Kruskal–Wallis *P*-value = 0.0025, U-test *P*-value = 1. Immediate memory, delayed memory and total index RBANS scores trended lower in the aMCI with spikes compared with no spike group, but the differences did not reach statistical significance in the context of the limited sample size.

There were no statistically significant cognitive differences between the aMCI groups with and without spikes (Table 2). Immediate memory, delayed memory and total index RBANS scores trended lower in the aMCI with spikes compared with no spike group, but the differences did not reach statistical significance in the context of the limited sample size (Fig. 4).

### Cortical thickness and subcortical volumes

We compared total intracranial volume, total cortical volume, total cortical grey and white matter volume, left and right amygdala volume and left and right hippocampal volume, all corrected for estimated total intracranial volume using FreeSurfer 6.0.0. There were no significant differences in regional or global cortical thickness and volume or subcortical regions of interest between aMCI and HC groups and between the aMCI with spikes and aMCI without spikes groups (Table 3).

**Table 3:** Neuroimaging

Regions of brain	aMCI with spikes (*n* = 6)	aMCI without spikes (*n* = 6)	Controls (*n* = 20)	*P*-value (Kruskal–Wallis)	*P*-value (U-test)
Left hippocampus	3516.9 ± 712.1	3357 ± 659.8	3740.9 ± 439	0.54	0.69
Left amygdala	1274.7 ± 282.6	1424.8 ± 314.1	1495 ± 173	0.19	0.69
Right hippocampus	3546.8 ± 758.1	3578.5 ± 800.5	3819 ± 331	0.77	1
Right amygdala	1623.8 ± 291	1661.8 ± 317	1808 ± 260	0.27	0.94
Cortex Vol	447 469.3 ± 27 726.4	433 179.1 ± 27 004	468 757 ± 46 399	0.40	0.59
Cortical white matter Vol	435 993.3 ± 57 845.8	421 761.6 ± 33 886.7	442 624 ± 66 472	0.51	0.69
Total grey Vol	602 604.1 ± 39 072.3	594 406.2 ± 34 474.9	632 624 ± 58 429	0.57	0.69

Furthermore, no significant differences were observed in cortical thickness and subcortical volumes among the three groups (aMCI with spikes, aMCI without spikes and HCs) when considering age, sex and education as covariates. The generalized linear model, which accounted for the non-normal distribution of the brain volume data, yielded *P*-values above the threshold for statistical significance (Supplementary Table 3 and Supplementary material).

## Discussion

The main finding of this exploratory study is that EEG spikes are reliably detected with overnight HD-EEG. In our cohort, they were present in 46% of patients with aMCI and in 10% of HC. The presence or absence of EEG spikes in aMCI patients was not associated with quantitative MRI metrics or neuropsychological performance. These findings could potentially aid in the early identification of specific disease phenotypes in the course of Alzheimer’s disease-related dementia.

Longer duration standard-density EEG (∼21 electrodes, >1 h) and MEG have both been used to capture EEG spikes in patients with Alzheimer’s disease with an incidence of around 20–40%.^24,25^ Vossel *et al.*^11^ have argued for a combination of MEG and low-density EEG as the standard because some patients only demonstrate spikes in one modality. HD-EEG (>64 electrodes) has the benefits of both standard EEG (longer monitoring periods and more cost-effective than MEG) and MEG (high-density sensor coverage particularly of the temporal lobes).

In this study, we examined 33 participants (13 with aMCI and 20 age-matched HCs), all of whom underwent neuropsychological testing, overnight HD-EEG and structural brain MRI. HD-EEG was reviewed blinded to diagnosis by two neurologists; 46% of aMCI subjects compared with 10% of controls had EEG spikes. The pivotal study of Vossel *et al*. showed a spike incidence of the combination of standard-density EEG (21%) with MEG (33%) that yields a combined incidence of 42.4% (12% had spikes on both) and 11% in controls. These rates are very similar to what we found in our cohort, even if there is a substantial disparity in spike frequency between the two studies: one to four spikes per night in our cohort compared with 0.03 to 5.18 per hour on LTM-EEG and 1 to 20 per hour on MEG in Vossel *et al*. Indeed, we must specify that some differences between the cohorts were detected. Vossel’s group was younger than our cohort, had more atypical presentations of Alzheimer’s disease, included early dementia and had more females. However, this HD-EEG study thus had a similar sensitivity to MEG and overnight standard-density EEG with the added benefit of being able to be performed in high-resolution source localization on all spikes detected. The spikes detected in this study were small amplitude, seen in slow-wave sleep, with a predominantly temporal lobe localization (Fig. 3).

Neuropsychological testing showed the aMCI group, as expected, to have impaired immediate and delayed memory compared with controls. However, within the aMCI group, there was no statistically significant difference in cognitive performance between those with and without spikes. That said, the limitation of this investigation is the modest sample size and statistical power, but a trend was noted for memory function in the ‘spike-positive’ group. There was also no statistical difference in overall grey matter volume, total white matter volume or hippocampal volume between those with spikes and those without.

Longitudinal studies that consider both the early and late stages of the disease progression, along with larger sample sizes, would greatly assist in providing a clearer understanding of the impact of EEG spikes on cognition. These studies would also help determine whether there is indeed a distinct functional and cognitive progression between phenotypes with and without epileptiform activity.

### Epileptiform phenotype of Alzheimer’s disease

None of the participants in this study had a history of overt clinical seizures. The vast majority of EEG spikes found were low-amplitude temporal spikes in NREM sleep. These low-amplitude spikes have been described by Lam *et al*.^8^ and overall have a lower association with clinical seizures than more typical ‘epileptiform spikes’ that have higher amplitude and after-going slow-wave components that are present outside of slow-wave sleep. The increased synchrony of slow-wave sleep makes EEG spikes higher amplitude and easier to detect.^9^ As would be expected, the more typical epileptiform spikes are more often associated with clinical seizures.

The clinical and pathogenic significance of these lower amplitude spikes therefore remains to be determined. There is some suggestion that they are not a benign epiphenomenon. Even if previous research in patients with focal epilepsy did not find a correlation between interictal discharge rates and neuropsychological scores, it is noteworthy that the same studies demonstrate that spatial and temporal dynamics of spike occurrences directly affect specific cognitive domains.^26,27^ Furthermore, in a randomized control trial administering patients with mild dementia [mean Clinical Dementia Rating (CDR) scale ratings of 3.8], a low dose of levetiracetam (125 mg twice a day) found that patients without a clinical history of seizures, but with EEG spikes, had resulting improvement in memory and executive function.^14^ Borrowing from the clinical experience of invasive EEG monitoring (i.e. depth electrodes placed directly into the hippocampus) in patients with epilepsy might explain some of these results. MEG and scalp EEG are relatively insensitive to highly localized EEG activity in the hippocampus. Most epileptic spikes in the hippocampus have no obvious scalp correlation. Beyond epileptiform discharges, there are even micro or subclinical seizures without clear clinical manifestation that are common with invasive EEG recordings from the hippocampus.^8,28,29^ Low-amplitude EEG spikes without a history of clinical seizures are sometimes referred to as subclinical.^24,30,31^ These discharges are not ‘subclinical’ as they may cause disruptions in memory and executive functioning as they are known to do in patients with epilepsy, especially in light of the recent results of the Vossel *et al.* study,^14^ with both short-term and long-term impacts.^10,32,33^

Several questions follow from these results. Is there really a distinct epileptiform phenotype of Alzheimer’s disease? In other words, is there a specific subgroup of Alzheimer’s disease patients with EEG abnormalities that respond to anti-seizure medications even without overt clinical seizures? If so, what is different about this group of patients from other Alzheimer’s disease patients?

Alzheimer’s disease patients with epileptiform abnormalities have an increased rate of cognitive decline and reduced survival.^12,13^ There is an increased risk of seizures in patients with early onset autosomal dominant Alzheimer’s disease with mutations in *APP* and *presenilin*,^34,35^ suggesting that amyloid deposition is required for expression of the epileptiform Alzheimer’s disease phenotype. Murine models of amyloid overexpression carrying the mutant human *APPswe* and *PS1dE9* genes have increased cortical hyperexcitability with a decrease in resting membrane potential and increased stimulus-induced excitatory post-synaptic potentials.^35^

Hence, the amyloid-β peptide toxicity is mediated by several mechanisms. In detail, the extracellular amyloid deposition increases the level of glutamate both in the synaptic cleft and around the extra-synaptic space, which in turn alters the level of excitability. These alterations cause increased long-term depression and reduced long-term potentiation; thus, the increased hyperexcitability is sustained by reduced inhibition by interneuron at the network level. Furthermore, the amyloid deposition is associated with abnormal tau phosphorylation and neurodegeneration.^6^ It seems mostly likely that increased cortical excitability is a downstream effect of tau deposition, since tau reduction in these animals can prevent spontaneous epileptic activity and seizures.^6,36-38^ The reasons why epileptiform activity is more pronounced in some patients may be related to genetic and environmental background and the location and type of tau accumulation. Moreover, for more than 10 years, it has been known from studies in mouse models and humans that Alzheimer’s disease-related pathology and epilepsy share the association with specific ApoE genotype. Indeed, it is speculated that an abnormal lipid traffic between neurons and astrocytes leads to the accumulation of lipids in the brain, specifically through lipid-accumulated reactive astrocytes, that can boost the neuropathological vicious circle.^39,40^

Additionally, the natural history of the epileptiform phenotype of Alzheimer’s disease is not known. The parallel of temporal lobe epilepsy with the epileptiform phenotype of Alzheimer’s disease may be informative. In the early stages of temporal lobe epilepsy, seizures primarily present with experiential symptoms including sensations like déjà vu rather than prominent motor manifestations. The non-convulsive nature of the seizures can make their clinical identification more challenging, especially considering the absence of scalp-level EEG spikes in early temporal lobe epilepsy.^28^ As the epilepsy progresses scalp level, EEG spikes become more frequent and with higher amplitude, and in parallel, there is an increase in the severity of the seizures with loss of awareness and convulsions. The epileptiform phenotype of Alzheimer’s disease may follow a similar pathway. Is the epileptiform phenotype of Alzheimer’s disease progressive as in some cases of temporal lobe epilepsy? Only further longitudinal studies will answer these questions.

### A model for epileptiform phenotype

**Figure 5:**
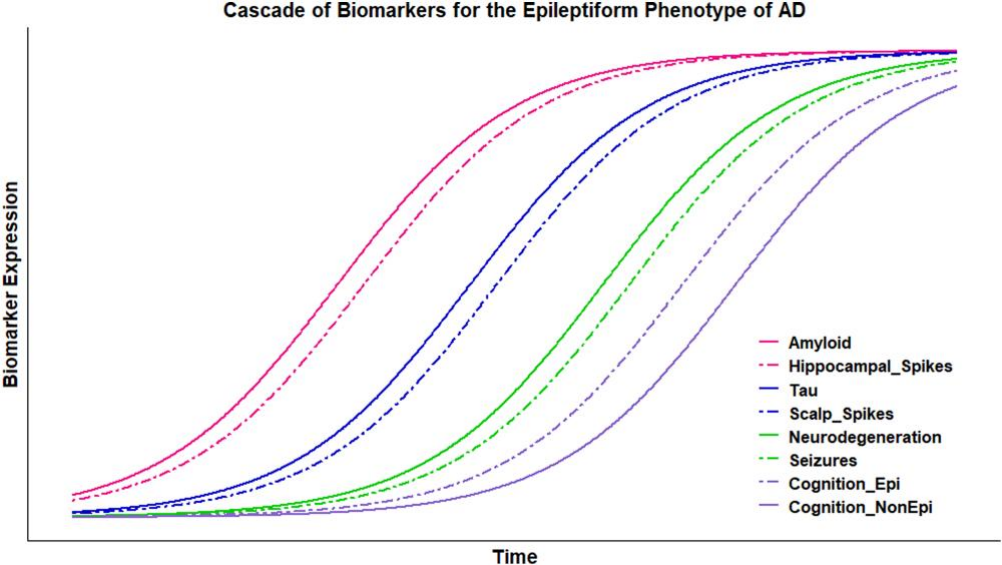
**A model for epileptiform phenotype.** The proposed dynamic relationship between epileptiform abnormalities (cortical hyperexcitability, epileptiform discharges and seizures) and the accumulation of amyloid, tau, neurodegeneration and cognitive deficits. Epileptiform discharges develop after amyloid deposition in the mesial temporal lobe and progress in parallel with hippocampal tau deposition, causing additional cognitive deficits. As tau deposition progresses, the scalp EEG spikes become evident and over time can become higher amplitude and, in some cases, overt clinical seizures can develop.

In Fig. 5, we propose a model of the epileptiform phenotype of Alzheimer’s disease.

In this model, cellular and molecular mechanisms underlying neuronal hyperexcitability^6^ occur only after hippocampal amyloid deposition. In a subsequent stage of the disease (Braak I and II),^41^ tau deposition in the mesial temporal lobe can initiate a positive feedback loop, consequently leading to the manifestation of epileptiform activity characterized by low-amplitude spikes that are difficult to identify on the scalp EEG. From animal studies, we know that neurodegeneration is not necessary for the appearance of epileptiform activity.^34,42,43^ As tau deposition progresses, the scalp EEG spikes become evident and over time can become higher amplitude and, in some cases, overt clinical seizures can develop. The EEG spikes cause additional cognitive deficits by disruption of hippocampal function. Fewer patients go on to have overt clinical seizures (10–22%)^3^ than have EEG spikes. The lack of overt clinical seizures in some patients with EEG spikes may stem from an incomplete expression of the epileptiform phenotype due to patient-specific factors or potentially from an under-detection of clinical seizures in dementia patients because of baseline cognitive difficulties and the likely lack of clinical awareness of the prevalence of seizures in this population. Potentially, there is another confounder. With disease progression, neurodegeneration in terms of atrophy, gliosis and synaptic loss does occur, as we know from morphological and functional studies.^44,45^ This results in a decrease in overall synaptic activity, which in turn leads to diminished synchronization essential for the expression of interictal epileptiform activity. Potentially, in the end stage of neurodegeneration, the regional neuro-circuity is degraded to such an extent that it can no longer produce epileptiform spikes or seizures. Paradoxically, this phenomenon ultimately gives rise to a state of cortical hypoexcitability.^46,47^

### Future directions

There is a need to determine whether the observed interictal discharges significantly impact cognition in the long term. A longer-term follow-up study is needed to address prospectively the nature, magnitude and course of impairment between patients with and without interictal discharges. Moreover, there are multiple exciting directions to pursue as the sample size grows including patterns in spectral power that could distinguish or characterize these populations and add a few other analytic directions. Numerous studies have been conducted on the changes in spectral power and synchronization within the brain and various regions of the cortex in prodromal Alzheimer’s disease, MCI and Alzheimer’s disease-related dementia.^48-50^ Exploring potential variations in these measurements between different subphenotypes (epileptiform versus non-epileptiform) and at different stages of the disease would hold significant value in the future.

### Limitations

There are several key limitations of this study. First, only 13 subjects with aMCI were enrolled, therefore, the small sample size may limit the power to detect cognitive and structural differences between spike-positive and spike-negative aMCI groups. Moreover, the control group had a small sample size too; this is particularly relevant also in light of the data concerning the spike frequency (10%), higher than expected—if compared with the data in the literature. Second, there was no biomarker staging for amyloid and tau. Third, this is a cross-sectional study without longitudinal follow-up. Larger longitudinal studies aimed at early-stage disease with concurrent tau and amyloid staging are needed to test the model proposed in Fig. 5.

We acknowledge that a limitation in the diversity of our study participants exists, as the majority (30 out of 33) of subjects identified as White and our population is almost entirely male (90%). It is important to recognize that a more diverse representation among participants can provide a broader perspective and enhance the generalizability of our findings.

The number of events per night is limited, warranting caution when interpreting these findings. We believe that confirmatory studies on the same population and longitudinal evaluations are needed to shed light on the heuristic value and the clinical implications of these results in the future.

Electronic source imaging has several limitations. The cortical surface that we used in BrainStorm software does not include hippocampus and other deep structures (i.e. amygdala and thalamus), because source localization from these subcortical structures using scalp HD-EEG is unreliable (depth, ‘closed field’ configurations). Although in principle surface files can be created and incorporated from these deeper structures, we chose to not include them with a caveat that any activity from hippocampus picked up on the scalp EEG will be imaged in nearby cortical structures included in the cortical surface model. Therefore, with this method, we can only conclude that the main source of the activity that we reconstructed from the scalp is within the temporal lobes and their position in the medio-lateral and antero-posterior axis.

These spikes may have still originated from the hippocampus but propagated sufficiently during NREM sleep to be detectable as low-amplitude spikes on scalp EEG. Dipole fitting can be used to localize deeper spiking generating regions but has limitations especially in patients with relatively rare low-amplitude spikes.

Eventually, we have no MEG data about our sample, and then we are not able to directly compare our data with other studies.

## Conclusion

Low-amplitude temporal EEG spikes are present in patients with aMCI with a prevalence similar to that reported in early-stage Alzheimer’s disease. Overnight HD-EEG appears to have a sensitivity comparable with MEG combined with overnight conventional EEG. Longitudinal studies looking at pre-morbid or early-stage Alzheimer’s disease are needed to characterize the epileptiform phenotype of Alzheimer’s disease and determine the optimal points of intervention and natural history.

## Supplementary Material

fcad302_Supplementary_Data

## Data Availability

The data that support the findings of this study are available from the corresponding author (R.C.) upon reasonable request.
