# Supplementary material for: Prevalence and localization of nocturnal epileptiform discharges in mild cognitive impairment
Source: Brain Commun. 2023 Nov 8;5(6):fcad302. doi: 10.1093/braincomms/fcad302 (PMC10642616; doi:10.1093/braincomms/fcad302)
Supplement: fcad302_Supplementary_Data [file fcad302_supplementary_data.zip › Supplementary Methods and Tables.docx]

**Supplementary Methods**

***Experimental Timeline***

As per the protocol, at the beginning of the visit (which lasted approximately 3 hours), we obtained written informed consent from the patients. Subsequently, we conducted screenings for depression, anxiety, and sleep disorders. Additionally, we gathered a detailed health history and conducted standardized neurocognitive testing. A geriatrician or geriatric nurse practitioner performed a physical examination, which included a neurological assessment, to identify any pre-existing sleep disorders, central neurological conditions, or risk factors for aMCI. To avoid test fatigue or distress, we allowed breaks as needed. Lastly, we collected a saliva sample to determine the APOE4 carrier status. Participants were instructed to maintain consistent bedtime schedules at their residences for a duration of two weeks preceding the sleep study. Following the two-week period, a structural MRI scan was conducted prior to the overnight sleep study. Before undergoing the MRI scan, the participants underwent an MRI screening process. Eventually, hd-EEG and PSG testing was conducted overnight at the Wisconsin Sleep Lab. The registration lasted on average eight hours.

***Power Calculation***

We conducted the power calculation to determine the number of patients required to achieve significance in the NPS tests for aMCI patients with and without spikes. We calculated the post-Hoc Effect size using the data of our cohort.

Based on our calculations, for Delayed Memory, the Effect size = 0.6957. Applying a one Tail Wilcoxon-Mann-Whitney test, with a Power of 0.90, we would need a total sample size of 78 (36 aMCI + vs 42 aMCI-) to reach a statistically significant difference.

For Immediate memory, the Effect size = 0.4195. Applying a one Tail Wilcoxon-Mann-Whitney test, with a Power of 0.90, we would need a total sample size of 208 (96 aMCI + vs 112 aMCI-) to reach a statistically significant difference.

The power analysis was performed using G*Power Software^1^, taking into consideration the effect size, desired power level, and significance level.

**Supplementary Tables**

**Supplementary Table 1: Temporal and spatial features of EEG spikes in patients and controls**

**Study ID Group spikes total Stage Source**

|  |
| --- |

01_2012_2125 aMCI 1 N2 LTMA

15_2013_0021 aMCI 1 N3 RTLA

21_2013_2211 HC 2 wake(2) LTLA

30_2014_2158 aMCI 3 N2(2), N3 RTLA, RTMA

33_2014 aMCI 3 N2(3) LTMA, RTA

36_2014_2311 aMCI 1 N2 LTMA

37_2014_2119 aMCI 1 N2 LTA

49_2015_2149 HC 2 N2(2) LTMA

LTMA = Left Temporal Mesial Anterior; RTLA = Right Temporal Lateral Anterior; LTLA = Left Temporal Lateral Anterior; RTMA = Right Temporal Mesial Anterior; RTA = Right Temporal Anterior; LTA = Left Temporal Anterior.

**Supplementary Table 2: Demographic features in patients with aMCI with and without spikes (aMCI + vs aMCI -)**

| **Characteristic** | **aMCI + aMCI -** | **p-value** |  |  |
| --- | --- | --- | --- | --- |
| Age, years | 73 ± 8 72 ± 8 | 0.8 |  |  |
| Education, years | 14.8 ± 3.3 14.7 ± 3.6 | 0.9 |  |  |
| Male sex n (%) | 5/6 (83) 6/7 (85) | 1 |  |  |
| ApoE ɛ4 carrier, n(%) | 4/6(67) 5/7 (71) | 1 |  |  |
|  |  |  |  |  |
| Race Indian native/Alaskan n(%) | 0/6(0) 1/7(15) | 1 |  |  |
|  |  |  |  |  |
| Race white n (%) | 6/6 (100) 6/7 (85) | 1 |  |  |
|  |  |  |  |  |
|  |  |  |  |  |
|  |  |  |  |  |
|  |  |  |  |  |
|  |  |  |  |  |
|  |  |  |  |  |

**Supplementary Table 3: Effect of age, sex and education on cortical and sub-cortical volumes**

| **Regions of the brain** | **Age Sex** | **Years of education** |  |  |
| --- | --- | --- | --- | --- |
| Left hippocampus | 0.3091 0.5397 | 0.3831 |  |  |
| Right hippocampus | 0.7613 0.9831 | 0.8372 |  |  |
| Left Amygdala | 0.9224 0.9273 | 0.8060 |  |  |
| Right Amygdala | 0.7140 0.9281 | 0.4854 |  |  |
|  |  |  |  |  |
| Cortex volume | 0.1050 0.4481 | 0.1327 |  |  |
|  |  |  |  |  |
| White matter volume | 0.1393 0.6461 | 0.1230 |  |  |
|  |  |  |  |  |
| Total grey volume | 0.2595 0.8558 | 0.2230 |  |  |
|  |  |  |  |  |
|  |  |  |  |  |
|  |  |  |  |  |

P-values for Kruskal-Wallis tests assessing the effects of age, sex, and education on brain areas in the study groups

**Supplementary reference**

1. Franz Faul, Edgar Erdfelder, A. B. & A.-G. L. Statistical power analyses using G*Power 3.1: Tests for correlation and regression analyses. *Behav. Res. Methods* **41**, 1149–1160 (2009).
