# Supplementary material for: Prevalence and localization of nocturnal epileptiform discharges in mild cognitive impairment
Source: Brain Commun. 2023 Nov 8;5(6):fcad302. doi: 10.1093/braincomms/fcad302 (PMC10642616; doi:10.1093/braincomms/fcad302)
Supplement: fcad302_Supplementary_Data [file fcad302_supplementary_data.zip › Supplemental figures_new.pdf]

Supplementary Figures

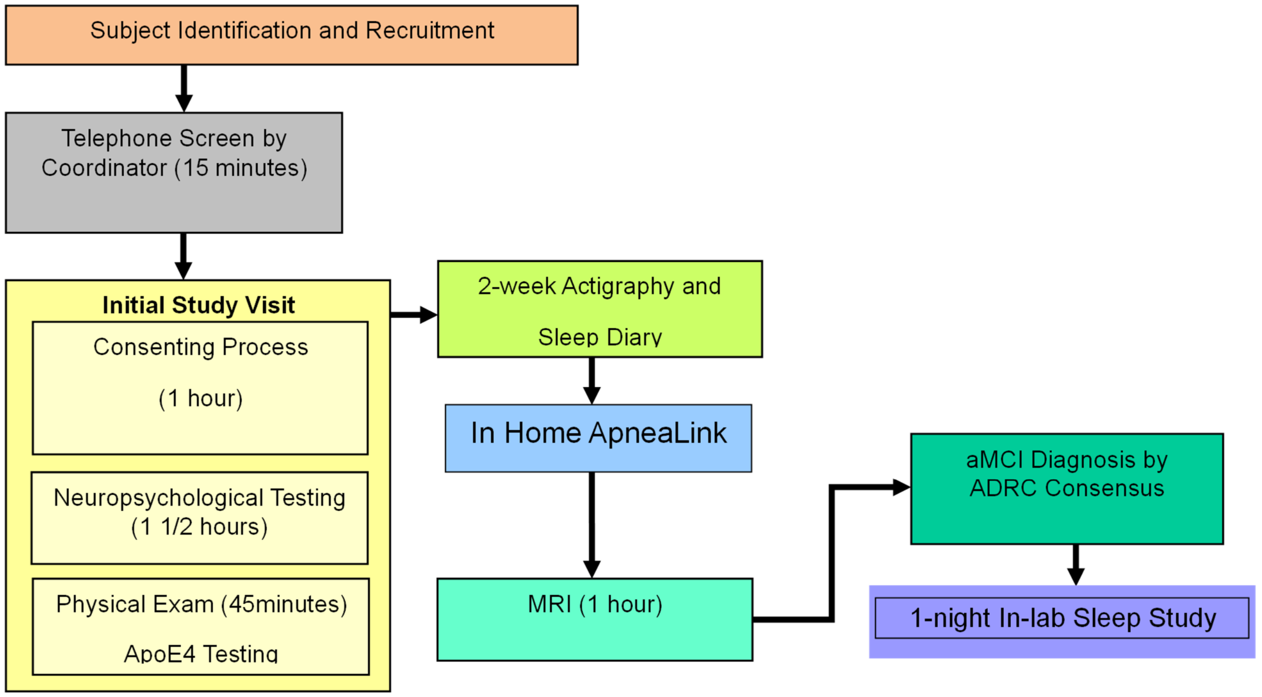

Figure S1. Experimental Timeline. As per the protocol, at the beginning of the visit (which lasted approximately 3 hours), we obtained written informed consent from the patients. Subsequently, we conducted screenings for depression, anxiety, and sleep disorders. Additionally, we gathered a detailed health history and conducted standardized neurocognitive testing. A geriatrician or geriatric nurse practitioner performed a physical examination, which included a neurological assessment, to identify any pre-existing sleep disorders, central neurological conditions, or risk factors for aMCI. To avoid test fatigue or distress, we allowed breaks as needed. Lastly, we collected a saliva sample to determine the APOE4 carrier status. Participants were instructed to maintain consistent bedtime schedules at their residences for a duration of two weeks preceding the sleep study. Following the two-week period, a structural MRI scan was conducted prior to the overnight sleep study. Before undergoing the MRI scan, the participants underwent an MRI screening process. Eventually, hd-EEG and PSG testing was conducted overnight at the Wisconsin Sleep Lab. The registration lasted on average eight hours.

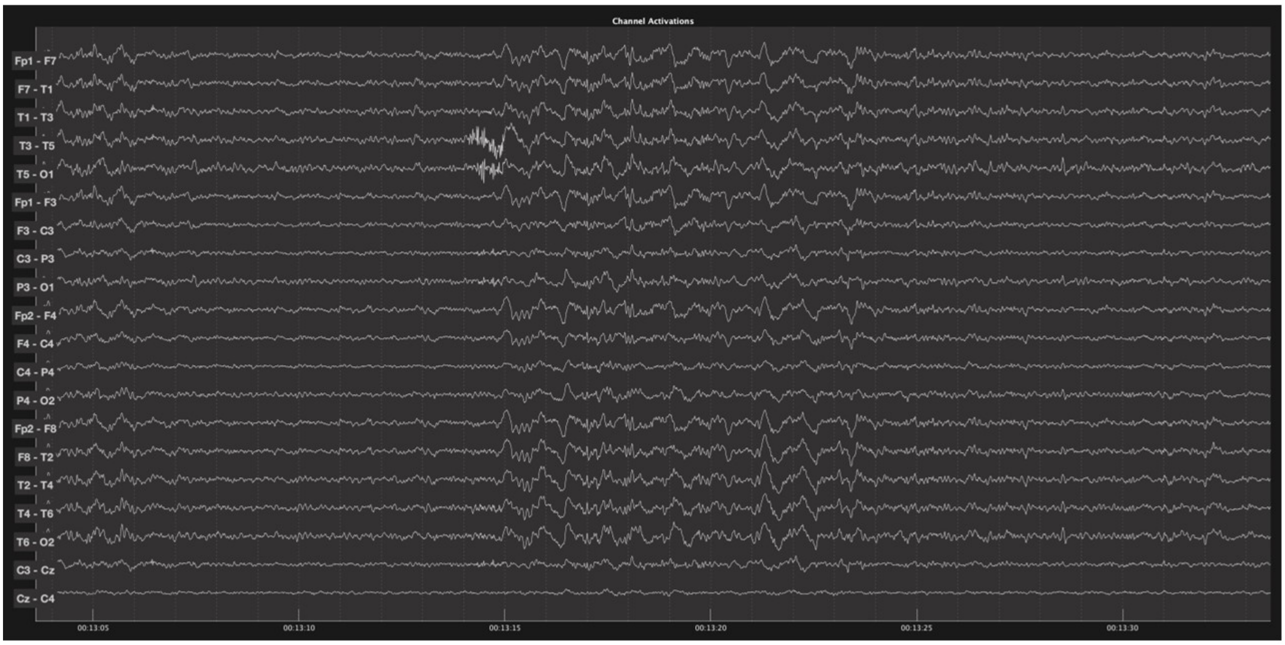

Figure S2. 30 seconds epoch during sleep in one subject with aMCI, showed with conventional 10-20 clinical montage. This serves as an illustrative example highlighting the good quality of the data.

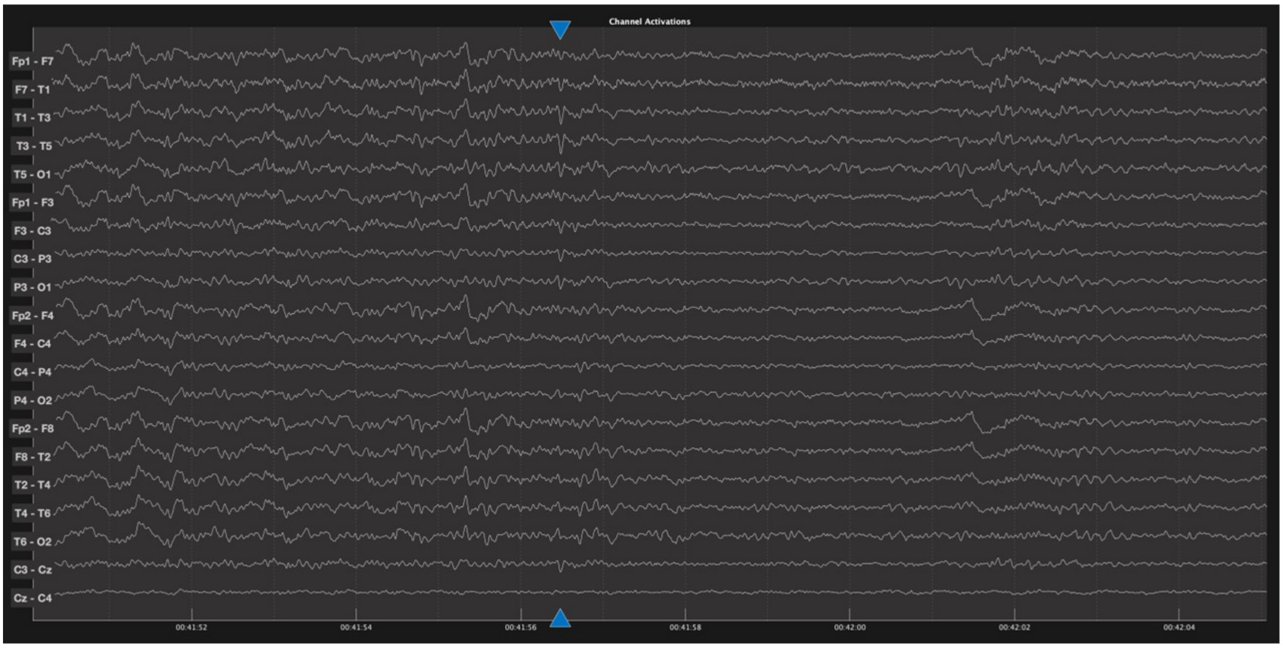

Figure S3. EEG data on 10 20 conventional montage showing a left temporal spike during sleep in a subject with aMCI, 20 seconds long. The arrows mark the EEG spike.

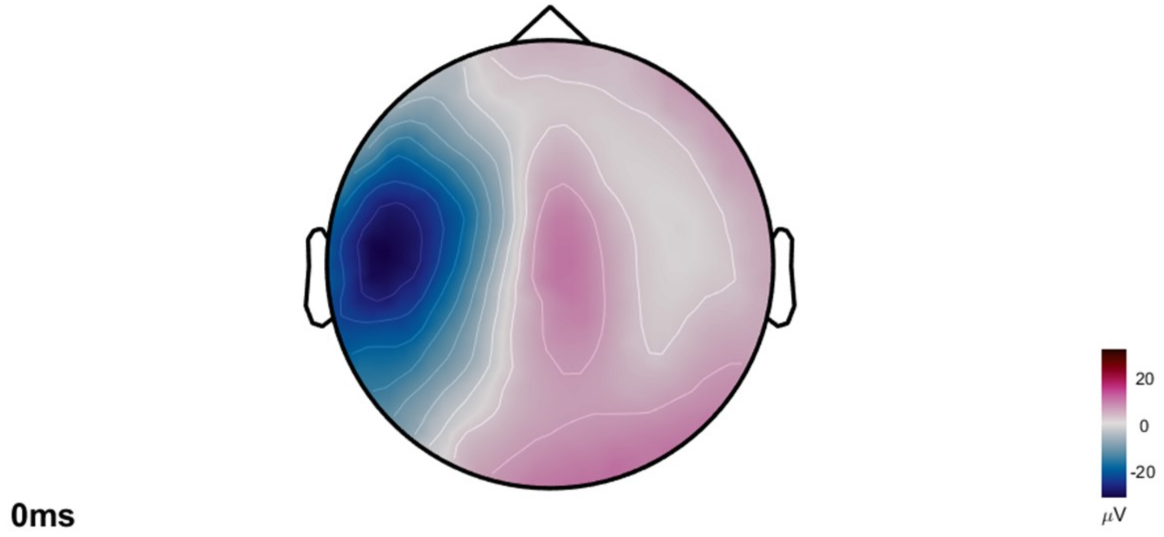

Figure S4. 2D Amplitude topography at time peak of the spike

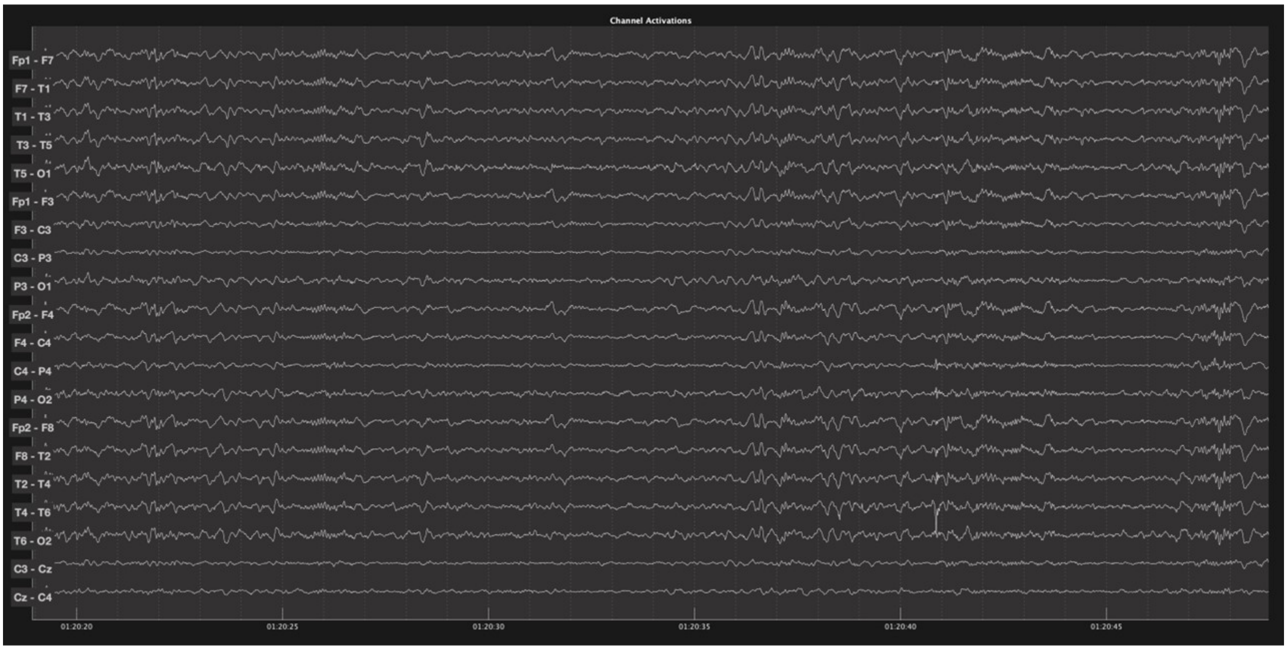

Figure S5. 30 seconds epoch during sleep in one healthy control, showed with conventional 10-20 clinical montage.

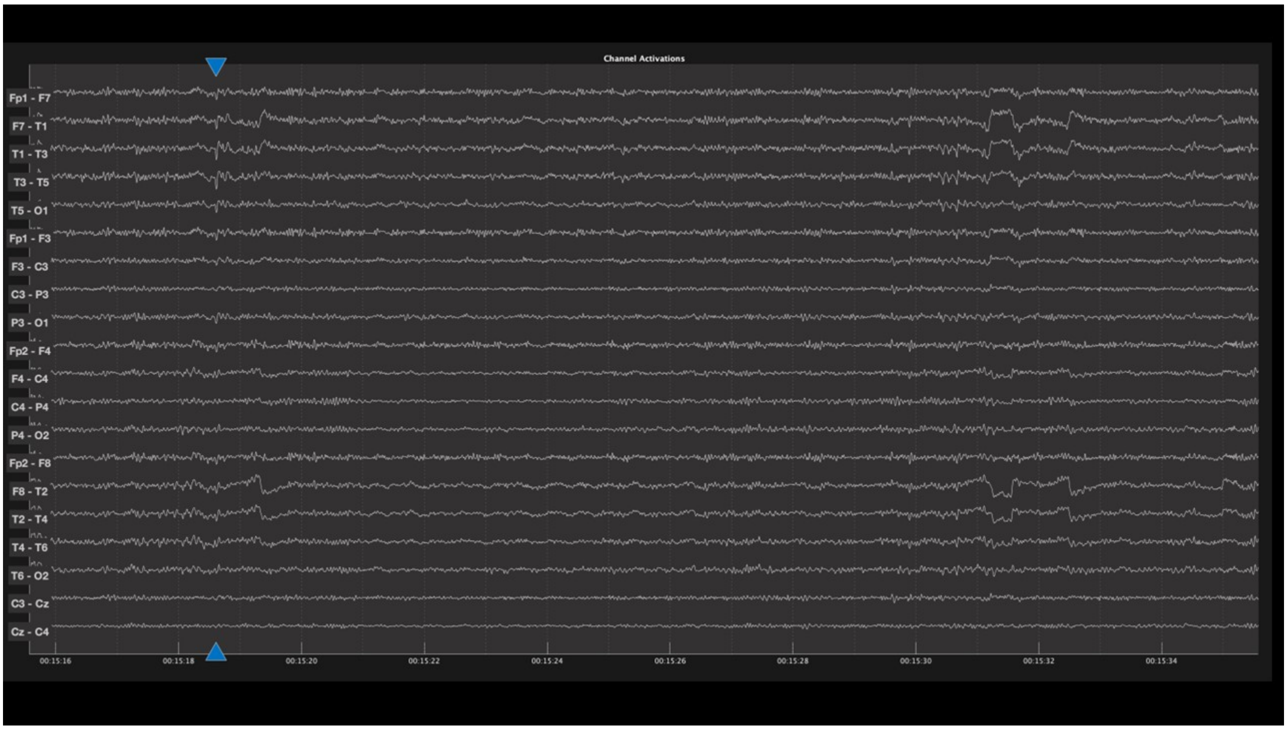

Figure S6. EEG data on 10 20 conventional montage showing a left temporal spike during sleep in one healthy control, 20 seconds long. The arrows mark the EEG spike.

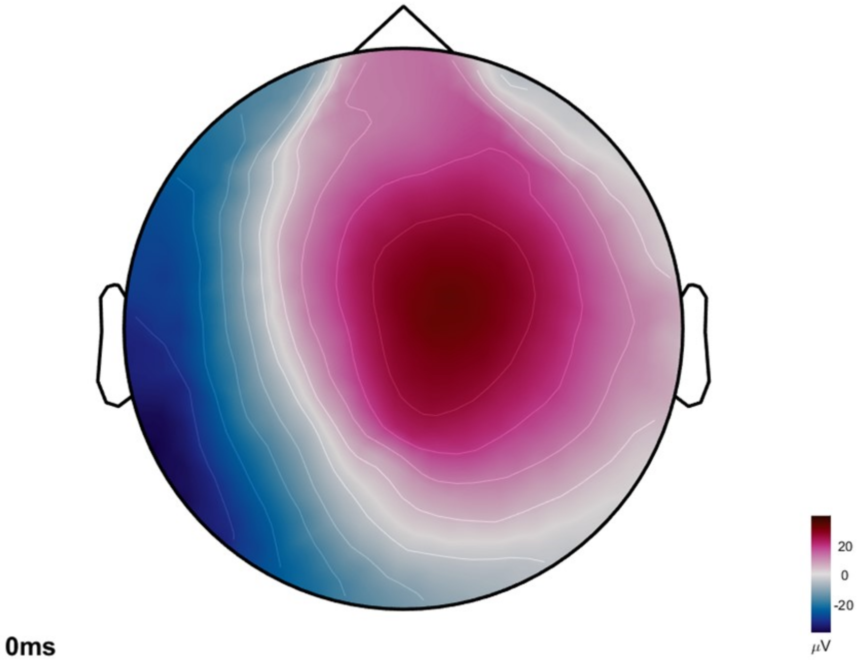

Figure S7. 2D Amplitude topography at time peak of the spike

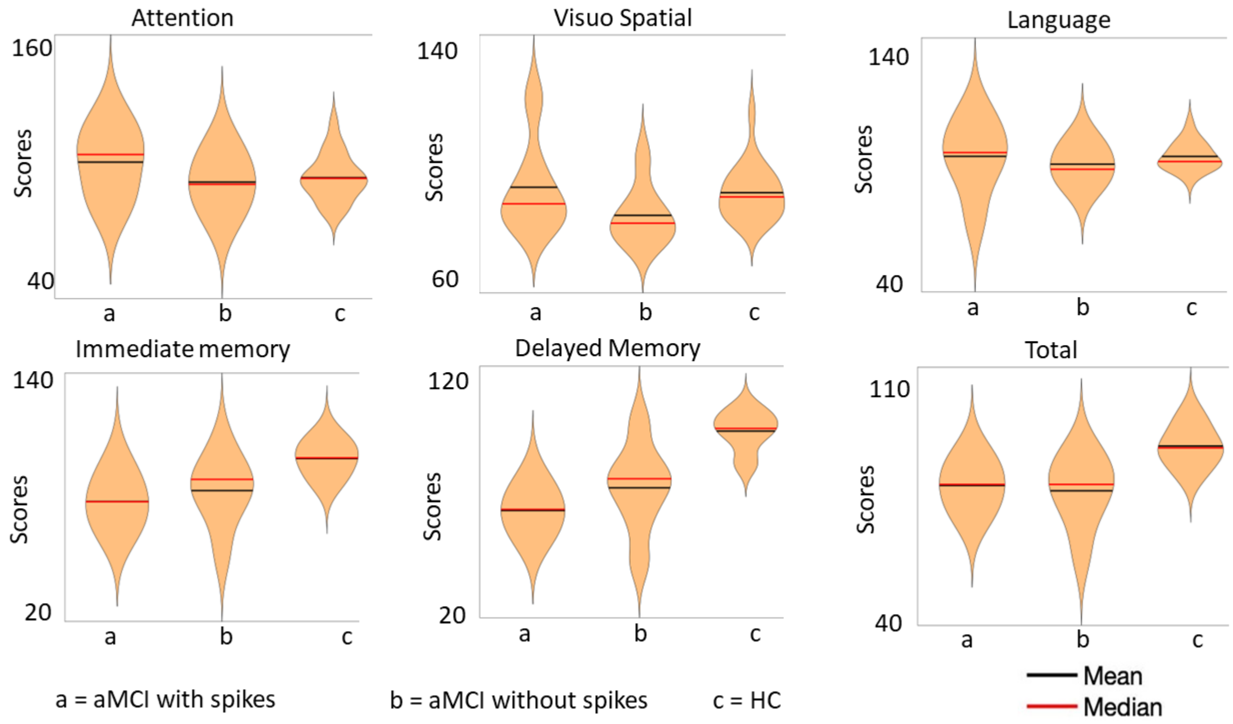

Figure S8 Neuropsychological assessment. Violin plots with scores comparison between patients with aMCI with spikes (violin plot 1), patients with aMCI without spikes (violin plot 2), and all HC (violin plot 3). On the Y axis the scores. From left to right, top to bottom Attention, Visuo-spatial skills, Language, Immediate memory, Delayed memory, Total RBANS score. No statistically significant differences were found between the aMCI groups with versus without spikes. Immediate memory, delayed memory, and total index RBANS scores trended lower in the aMCI with spikes compared to no spike group, but the differences did not reach statistical significance in the context of the limited sample size. aMCI = amnestic Mild Cognitive Impairment. HC = Healthy Controls. RBANS = Repeatable Battery for the Assessment of Neuropsychological Status.
